# A Compend of Human Physiology

**Published:** 1899-10

**Authors:** 


A Compend of Human Physiology. Especially adapted for the Use of Medical Students. Quiz Compends, No. 4. By Albert P. Brubaker, A. M., M. D., Adjunct Professor of Physiology and Hygiene in the Jefferson Medical College; Professor of Physiology in the Pennsylvania College of Dental Surgery, etc., etc. Ninth edition, with new illustrations. Philadelphia: P. Blakiston's Son & Co. 1899.

This volume belongs in the well known seiies of quiz compends, published by Blakiston. It is not written in form of question and answer, as some other of the series are—a style which seems to the reviewer decidedly objectionable. This edition has been revised and enlarged and will doubtless continue in favor with a large number of medical students. M. J. F.

